# Krampfanfall mit intrakraniellen Blutungen nach Sectio caesarea in Spinalanästhesie

**DOI:** 10.1007/s00101-022-01203-x

**Published:** 2022-09-27

**Authors:** Ben Thewes, Nils Christian Lehnen, Felix Bode, Franziska Dorn, Brigitte Strizek, Anna Katharina Diedrich, Tobias Hilbert

**Affiliations:** 1 Klinik für Anästhesiologie und Operative Intensivmedizin, Universitätsklinikum Bonn, Venusberg-Campus 1, 53127 Bonn, Deutschland; 2 Klinik für Neuroradiologie, Universitätsklinikum Bonn, Bonn, Deutschland; 3 Klinik für Neurologie, Universitätsklinikum Bonn, Bonn, Deutschland; 4 Abteilung für Geburtshilfe und Pränatale Medizin, Zentrum für Geburtshilfe und Frauenheilkunde, Universitätsklinikum Bonn, Bonn, Deutschland

## Falldarstellung

### Anamnese

Eine 26-jährige Zweitgravida (Para 0) stellt sich nach bisher unauffälligem Schwangerschaftsverlauf bei Geminigravidität zur elektiven Sectio in der 37 + 0. Schwangerschaftswoche vor. Vorerkrankungen, Allergien oder eine relevante Dauermedikation bestehen nicht. Ein anästhesiologisches Vorgespräch war bereits 5 Wochen vor dem geplanten Eingriff erfolgt. Die Laboruntersuchungen ergaben Normalbefunde. Am Tag des Eingriffs wiegt die Patientin 79 kg (vor Schwangerschaft 58 kg) bei einer Körpergröße von 167 cm. Nach Etablierung des Routinemonitorings wird unter Co-Hydratation mit 1000 ml Kristalloid eine Spinalanästhesie mit einer 25-G-Spinalkanüle auf Höhe L3/L4 in sitzender Position problemlos durchgeführt (Barbotage unauffällig, Injektion von 8,5 mg Bupivacain hyperbar und 5 μg Sufentanil). Unmittelbar nach dem Wechsel in Linksseitenlage gibt die Patientin starke bifrontale Kopfschmerzen an. Eine mäßige Hypotonie und Sinusbradykardie (112/74 mm Hg, 57 bpm) werden mit Atropin (0,5 mg) und Theodrenalin/Cafedrin (4/80 mg) behandelt. Nach Aufsteigen der sensomotorischen Blockade bis auf Höhe Th6 binnen 14 min erfolgt der Schnitt. Drei bzw. vier Minuten später werden zwei gesunde Mädchen entwickelt. Der weitere Verlauf gestaltet sich problemlos, die Kopfschmerzen der Patientin bestehen jedoch fort und werden mit Paracetamol behandelt. Nach 45-minütiger Operationsdauer wird die Patientin kreislaufstabil und ohne weitere neurologische Auffälligkeiten in den Kreißsaal zurückverlegt.

### Klinischer Befund

Drei Stunden nach dem Eingriff beklagt die Patientin Kribbelparästhesien im rechten Arm bei zunächst unbeeinträchtigter Motorik und Temperaturempfindung. Die Vitalwerte sind unauffällig. Eine Stunde später zeigen sich zusätzlich motorische Auffälligkeiten im Sinne eines Tremors der rechten Hand. Die Patientin ist dabei wach und zu allen Qualitäten orientiert, die Kopfschmerzen sind gebessert. Eine Restwirkung der Spinalanästhesie kann nicht mehr festgestellt werden. Die Pupillen sind isokor, mittelweit und beidseits direkt sowie konsensuell lichtreagibel.

Plötzlich kommt es zu tonisch-klonischen Entäußerungen, beginnend in der rechten oberen Extremität, welche jedoch rasch in einen generalisierten Grand-Mal-Anfall mit Blickdeviation nach oben bei weit geöffneten Augen, Hypersalivation und einer deutlichen Lippenzyanose übergehen. Unverzüglich erfolgen die Gaben von 5 mg Diazepam sowie 5 g Magnesium i.v., woraufhin der Anfall nach ca. 90 s sistiert. Die Patientin zeigt sich nun postiktal deutlich verhangen und hypertensiv entgleist (160/85 mm Hg). Es wird eine Magnesiumdauerinfusion (1 g/h) begonnen.

### Diagnose

**Figure 1:**
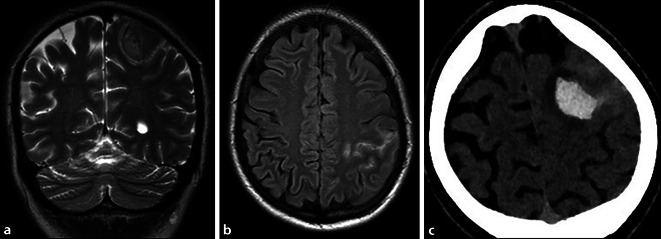

Die Patientin wird zur weiteren Beurteilung in das Neuro-Zentrum verlegt. Nach Ankunft treten erneut tonisch-klonische Entäußerungen des rechten Armes auf, welche nach Diazepam und Levetiracetam sistieren. Die kraniale MRT zeigt eine frische Parenchymblutung (ICB) links parietal mit angrenzend geringem fokalem Hirnödem sowie eine unmittelbar angrenzende Subarachnoidalblutung (SAB) ohne sichere Blutungsquelle (Abb. 1a, b). Während der Überwachung auf der Stroke Unit gibt die Patientin noch leichte Kopfschmerzen an, außerdem zeigt sich eine intermittierende Schwäche des rechten Armes mit Absinken im Arm-Halte-Versuch. In einer Verlaufs-CT am Folgetag kommen die im Wesentlichen konstante Parenchymblutung und eine diskrete Umverteilung der vorbestehenden SAB-Komponente zur Darstellung (Abb. 1c). Ein ähnlicher Befund zeigt sich 2 Tage später in der Verlaufs-MRT.

### Verlauf

**Figure 2:**
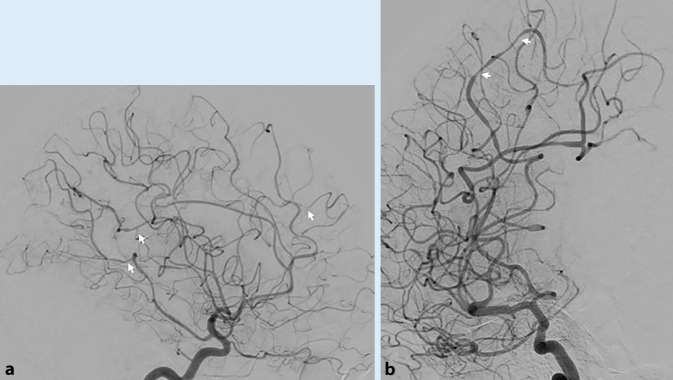

In den folgenden Tagen treten intermittierend rechtsseitige Gesichtsfeldausfälle sowie Hypästhesien des rechten Armes auf. Angiographisch kommen deutliche Kaliberschwankungen peripherer Äste der Aa. cerebri media und posterior beidseits zur Darstellung, vergleichbar einer primären ZNS-Vaskulitis („primary angiitis of the CNS“, PACNS) oder einem reversiblen zerebralen Vasokonstriktionssyndrom (Abb. 2a, b).

Nach 5 Tagen kann die Patientin mit der Empfehlung zu einer Reha-Maßnahme entlassen werden. Es persistiert eine latente Parese des rechten Armes mit Störungen der Sensibilität und Feinmotorik der Hand. Die antikonvulsive Therapie mit Levetiracetam wird fortgeführt. Laborchemisch ergeben sich keine Hinweise auf eine Vaskulitis. Drei Monate später berichtet die Patientin noch über ein leichtes Verschwommensehen auf dem rechten Auge sowie über intermittierende Kribbelparästhesien der rechten Hand. Die MRT zeigt eine regelrechte Gefäßkonfiguration ohne Nachweis eindeutiger Kaliberschwankungen bei weitestgehender Resorption der Blutungen. Aufgrund der Befundregredienz wird auf eine erneute diagnostische Angiographie verzichtet.

## Diskussion

Beim reversiblen zerebralen Vasokonstriktionssyndrom (RCVS oder früher Call-Fleming-Syndrom) kommt es durch reversible segmentale und multifokale Vasokonstriktionen der zerebralen Arterien zu stärksten Kopfschmerzen und u. U. weiteren neurologischen Defiziten wie Krampfanfällen, Schlaganfällen oder Subarachnoidalblutungen. Frauen sind mindestens doppelt so häufig betroffen wie Männer. Das Syndrom tritt typischerweise im mittleren Lebensalter mit einem Peak um das 42. Lebensjahr auf, allerdings reicht die Spanne vom Kindesalter bis jenseits des 70. Lebensjahres [12]. Ein RCVS kann spontan auftreten, in bis zu 60 % lässt sich aber ein Trigger eruieren. Häufigste Auslöser sind Schwangerschaft bzw. die postpartale Phase – in zwei Drittel der Fälle tritt das RCVS in der ersten Woche nach der Entbindung auf. Es existieren Fallberichte zu koinzidentem Auftreten von RCVS und Eklampsie [14]. Auch bestimmte Vorerkrankungen (Phäochromozytom, Porphyrie) oder neurochirurgische Operationen können prädisponieren [4], ebenso vasoaktive Substanzen wie Amphetamine, Cannabis, Kokain, Triptane und Antidepressiva [1, 3, 9, 10]. Zusammenhänge mit einer vorbestehenden Migräne werden in bis zu 42 % beschrieben [13]. Gerade das zeitgleiche Auftreten zusammen mit der Gabe kreislaufwirksamer Triggersubstanzen wie Phenylephrin oder Atropin, welche häufig im Rahmen geburtshilflicher Eingriffe gegeben werden, kann mit zur gesteigerten Prävalenz in diesem Patientenkollektiv beitragen und sollte zu entsprechender Aufmerksamkeit gemahnen [10].

Der klinische Beginn ist meist dramatisch. Bei mehr als 80 % der Patienten manifestiert sich das RCVS durch einen akuten Vernichtungskopfschmerz („Donnerschlagkopfschmerz“), der auch zur differenzialdiagnostischen Abgrenzung gegenüber einer PACNS dienen kann (welche eher schleichend mit dumpf-drückenden Schmerzen beginnt) [1]. Dieser bildet sich üblicherweise binnen 1–3 h zurück, persistiert danach meist als leichter Kopfschmerz im Hintergrund [12]. Die Hauptkomplikation stellen ischämische oder hämorrhagische Schlaganfälle in variabler Häufigkeit (9–63 %) dar. Häufig treten manifeste neurologische Begleiterscheinungen wie Verschwommensehen, Aphasie, Bewusstseinseintrübungen, Hemiparese und Tremor hinzu. Generalisierte epileptische Anfälle wie im vorliegenden Fall sind eher selten (1,2–5 %) [13]. Diagnostisch steht die zerebrale Bildgebung im Vordergrund. Da diese initial negativ sein kann, sollte sie bei Verdacht auf ein RCVS zeitnah wiederholt werden. In bis zu 80 % finden sich Parenchymläsionen, in 30–34 % kortikale subarachnoidale Blutungen [3]. In einer Fallserie von 18 Patientinnen traten intrakranielle Blutungen in 39 % der Fälle auf, vasogene Ödeme und Infarkte in jeweils 35 % der Fälle [5], auch von Parenchymblutungen wurden in Einzelfällen berichtet [6]. Die Kaliberveränderungen der zerebralen Gefäße, die sich von distal nach proximal hin ausbreiten, sind pathognomonisch und für die Diagnosestellung erforderlich. Diese gilt jedoch erst dann als gesichert, wenn die Reversibilität der Vasokonstriktion innerhalb von 3 Monaten angiographisch nachgewiesen werden kann [2, 4, 7]. Weitere Fallberichte schildern ebenfalls eindrucksvoll das klinische Bild mit stärksten Kopfschmerzen, Krampfanfällen und intrakranieller Blutung auf dem Boden einer (vorübergehenden) Deregulation des zerebralen Gefäßtonus im peripartalen Setting [8].

Aufgrund der relativen Seltenheit liegen zur Therapie des RCVS keine Daten kontrollierter Studien vor. Therapeutische Ansätze beinhalten v. a. die Unterbindung der auslösenden Triggerfaktoren nach Möglichkeit, die symptomatische Behandlung der Kopfschmerzen, die Vermeidung von Blutdruckentgleisungen sowie eine Therapie mit Nimodipin (Kalziumkanalblocker, Off-label-Gebrauch gemäß Anwendung bei einer aneurysmatischen SAB) [1, 13]. Für Letztere existieren auch Berichte zu positiven Verläufen im Falle der Koinzidenz mit einer Eklampsie, in der Präpartalphase sind jedoch immer die Auswirkungen auf den fetalen Kreislauf zu beachten [14]. Empfohlen wird die Überwachung am Monitor. Die Gabe von Glukokortikoiden, wie sie bei (differenzialdiagnostisch infrage kommenden) primär inflammatorischen Erkrankungen empfohlen wird, ist unbedingt zu vermeiden, da diese ein RCVS deutlich verschlechtern können [11]. Die Langzeitprognose hängt von möglicherweise bleibenden Einschränkungen in den Alltagsaktivitäten ab. Glücklicherweise erleiden weniger als 10 % der Betroffenen ein permanentes neurologisches Defizit [10].

## Fazit für die Praxis

Das RCVS betrifft häufig schwangere Frauen mittleren Alters. Charakteristisch ist die Kombination aus plötzlichem, heftigstem Kopfschmerz, fluktuierenden neurologischen Defiziten wie Krampfanfällen und reversiblen Vasokonstriktionen zerebraler Gefäße mit hämorrhagischen oder ischämischen Komplikationen. Differenzialdiagnostisch kommen eine Eklampsie oder auch eine Vaskulitis (PACNS) in Betracht, letztere zeigt in der Regel jedoch einen pathologischen Liquorbefund. Weitere Differenzialdiagnosen mit weitreichenden therapeutischen Konsequenzen (aneurysmatische SAB, Sinus- oder Hirnvenenthrombose, Karotisdissektion) kann eine Bildgebung zeitnah weitestgehend ausschließen. Bis dahin sollte eine Therapie mit Magnesiumsulfat in Absprache mit der Geburtshilfe eingeleitet werden, da RCVS und Eklampsie koinzident vorliegen können, selbst wenn typische Zeichen (Ödeme, art. Hypertonie, Proteinurie) fehlen. Von besonderer Bedeutung sind die zügige Diagnostik sowie die gute interdisziplinäre Zusammenarbeit.
